# Survival-Associated Metabolic Genes and Risk Scoring System in HER2-Positive Breast Cancer

**DOI:** 10.3389/fendo.2022.813306

**Published:** 2022-05-19

**Authors:** Chundi Gao, Huayao Li, Chao Zhou, Cun Liu, Jing Zhuang, Lijuan Liu, Changgang Sun

**Affiliations:** ^1^ College of First Clinical Medicine, Shandong University of Traditional Chinese Medicine, Jinan, China; ^2^ College of Basic Medical, Shandong University of Traditional Chinese Medicine, Jinan, China; ^3^ Department of Oncology, Weifang Traditional Chinese Hospital, Weifang, China; ^4^ Academy of Chinese Medical Sciences, Shandong University of Traditional Chinese Medicine, Qingdao, China; ^5^ College of Traditional Chinese Medicine, Weifang Medical University, Weifang, China

**Keywords:** HER2-positive breast cancer, metabonomics, prognostic risk scoring system, lasso cox regression analysis, survival prediction

## Abstract

Human epidermal growth factor receptor 2 (HER2)-positive breast cancer and triple-negative breast cancer have their own genetic, epigenetic, and protein expression profiles. In the present study, based on bioinformatics techniques, we explored the prognostic targets of HER2-positive breast cancer from metabonomics perspective and developed a new risk score system to evaluate the prognosis of patients. By identifying the differences between HER2 positive and normal control tissues, and between triple negative breast cancer and normal control tissues, we found a large number of differentially expressed metabolic genes in patients with HER2-positive breast cancer and triple-negative breast cancer. Importantly, in HER2-positive breast cancer, decreased expression of metabolism-related genes *ATIC*, *HPRT1*, *ASNS*, *SULT1A2*, and *HAL* was associated with increased survival. Interestingly, these five metabolism-related genes can be used to construct a risk score system to predict overall survival (OS) in HER2-positive patients. The time-dependent receiver operating characteristic (ROC) curve analysis showed that the predictive sensitivity of the risk scoring system was higher than that of other clinical factors, including age, stage, and tumor node metastasis (TNM) stage. This work shows that specific transcriptional changes in metabolic genes can be used as biomarkers to predict the prognosis of patients, which is helpful in implementing personalized treatment and evaluating patient prognosis.

## Introduction

Nearly 1.7 million new cases of breast cancer are reported worldwide every year (1). However, while the incidence continues to rise, breast cancer-specific mortality has fallen sharply—in part due to earlier detection, and in part due to improved adjuvant therapies (2). Increasing evidence indicates that metabolic changes may be potential biomarkers and therapeutic targets for cancer and can be of great significance in predicting outcomes and guiding treatment. Metabolic changes are one of the important characteristics of tumors. To maintain continuous proliferation, tumor cells must adjust their metabolism and nutrient acquisition methods, playing an important role in maintaining intracellular homeostasis and responding to intracellular and extracellular stimuli (3). Recent studies have shown that metabolic reprogramming, including changes in lipid metabolism, is manifested in various types of cancer, including breast cancer (4).

Metabolic pathways are tightly intertwined with cellular signals and epigenetic networks. Due to the heterogeneity of breast cancer and the diversity of molecular subtypes, the molecular mechanisms of different subtypes of breast cancer are different (5). From a molecular perspective, HER2-positive and triple-negative breast cancer have their own characteristic inheritance, epigenetics, and protein expression profiles (6). HER2-positive breast cancer has a better prognosis, which is mainly due to the effective treatment of HER2-positive breast cancer with surgery, radiotherapy and chemotherapy, endocrine therapy, molecular targeted therapy, and other treatments (7). Identification of biomarkers and cellular pathways that lead to poor prognosis can lead to the development of new effective therapies for those who do not respond to current treatments.

The reprogramming of cellular metabolism is one of the hallmarks of breast cancer. Breast cancer cells reshape the metabolic network to maintain their transformed state and survive in the harsh tumor microenvironment in ways such as promoting tumor blood vessel formation and destroying tumor immunity (8). Studies have reported that crosstalk between estrogen signaling elements and several key metabolic regulators alters the metabolism of breast cancer cells and reshapes the tumor metabolic network to accommodate poor perfusion, transient nutritional deficiencies, and added acidity (9). The relationship between cancer and metabolic pathways may reveal novel biomarkers and therapeutic targets. Targeting tumor metabolism is a potentially effective treatment for inhibiting breast cancer. Examining the metabolic phenotype between HER2-positive and triple-negative breast cancers can identify targeted metabolic changes and potential new treatment options. Identifying multiple metabolic targets for a cancer type from a large data set containing a large amount of tumor information, such as the Cancer Genome Atlas (TCGA), is an ideal step for selecting or generating useful anti-metabolic cancer therapies (10).

## Methods

### Data source

The TCGA database (https://cancergenome.nih.gov/) (11) provides data on mRNA expression and clinical information related to HER2-positive and triple-negative breast cancer. All the data needed for this study was downloaded from the TCGA data download window GDC (https://portal.gdc.cancer.gov/), including 161 cases of HER2-positive breast cancer samples, 115 cases of triple-negative breast cancer samples, and 34 normal samples. TCGA is used as a public open database, and the relevant information retrieved from it does not require further ethical approval.

### Analysis of mRNA Expression and Extraction of Metabolism-Related Genes

The Molecular Signatures Database (MSigDB) is a collection of annotated gene sets for use with Gene Set Enrichment Analysis (GSEA) software (https://www.gsea-msigdb.org/gsea/downloads.jsp) (12). From this website, we downloaded the background pathway gene set. Using R, we extracted the metabolic pathway in the background of GSEA and compared the genes in the pathway with the related genes in HER2-positive and triple-negative breast cancers, further obtaining the metabolic genes related to HER2-positive and triple-negative breast cancers.

## mRNA expression comparisons and pathway enrichment analysis

Using the edgeR software package, the metabolism-related gene expression data of HER2-positive breast cancer, triple-negative breast cancer, and normal samples were standardized, and the differences were analyzed to obtain abnormal metabolism-related mRNA. In addition, in order to understand the functional abnormalities caused by abnormal expression of metabolic genes in patients with HER2-positive breast cancer and triple-negative breast cancer, and to explore the possible biological processes and possible pathways, the functional enrichment analysis of gene expression was carried out by using the DAVID (http://david.abcc.ncifcrf.gov/) database (13). 0.05 was set as a cut-off condition for screening enrichment analysis of Kyoto encyclopedia of genes and genomes.

### Survival Analysis and Construction of the Prognostic Risk Scoring System

Using the survival software package and Cox proportional hazard model, the metabolism-related mRNA of HER2-positive breast cancer was analyzed by univariate analysis, and a survival curve was drawn. The genes with P<0.01 as a cutoff condition were of great significance to HER2-positive prognosis. Then, least absolute shrinkage and selection operator (lasso) penalized Cox regression analysis (14) was used to further screen it as an independent metabolic factor for predicting the prognosis of HER2-positive breast cancer. The complexity of the minimum absolute contraction and the lasso regression of the selection operator is controlled by the coefficient lambda. Using the cross-validation program cv.glmnet to determine the lambda value with minimum error to further reduce the number of prognosis-related metabolic mRNAs selected by univariate COX analysis, the risk scoring system can be constructed.

The formula for constructing a risk scoring system is as follows:


$$\text{Risk score }=\sum_{i=1}^{N}Exp_{i}\times \beta_{i}$$

β represents the coefficient, while Exp represents the expression value of metabolism-related mRNA. Taking the median risk score as the critical value, patients with HER2-positive breast cancer were divided into low risk and high risk groups. The survival rates of each group were compared by the Kaplan-Meier method and log-rank method.

### Comparison of Predictive Ability of Risk Scoring System Combined With Clinical Factors

In this study, in order to further compare the predictive performance of our HER2-positive breast cancer risk scoring system constructed by metabolic mRNA and evaluate whether it can be independent of other clinical parameters, univariate and multivariate analysis was conducted on the clinical data of HER2-positive breast cancer patients, including age, stage, TNM stage, survival time, and survival status. P<0.05 was considered to be statistically significant.

## Results

### Expression of Metabolism-Related Genes Was Altered Between HER2-Positive Breast Cancer, Triple-Negative Breast Cancer, and Normal Control Samples From the TCGA Database

**Figure 1:**
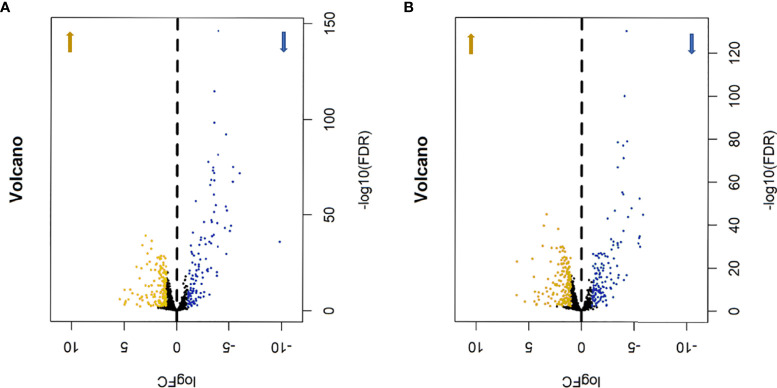
The volcano map of differentially expressed metabolism-related genes, Khaki represents up-regulated metabolic genes, blue represents down-regulated metabolic genes. **(A)** HER2-positive breast cancer, 154 up-regulated genes and 121 down-regulated genes. **(B)** triple-negative breast cancer, 181 up-regulated genes and 139 down-regulated genes.

In order to determine the difference in the expression of metabolic genes, we first compared the expression of mRNA in HER2-positive and triple-negative breast cancer samples with the genes of metabolism-related pathways in GSEA, so as to obtain metabolism-related genes. Then, the Edger software package was used to extract and analyze the differentially expressed data. Using | LogFC | >1 and P<0.01 as filter cutoff criteria, a total of 275 metabolic genes were obtained in HER2-positive breast cancer samples, including 154 up-regulated and 121 down-regulated (**Figure 1A**), while 320 metabolic genes were obtained in triple-negative breast cancer samples, including 181 up-regulated and 139 down-regulated (**Figure 1B**). In the cohort of difference analysis, we downloaded a total of 1096 breast cancer samples, including 161 HER2-positive breast cancer samples and 115 triple-negative breast cancer samples, which HER2-positive breast cancer accounted for 14.7% of the total and triple-negative breast cancer accounted for 10.5% of the total, and 34 cases of normal control samples. After removing the samples with incomplete prognostic information, the remaining 156 HER2-positive samples were used to construct the metabolism-related gene risk score model.

### Analysis of Characteristic Metabolic Pathways in HER2 Positive Breast Cancer and Triple Negative Breast Cancer

In order to further understand the characteristic metabolic pathways of HER2 positive breast cancer and triple negative breast cancer, the differential metabolism-related genes of the two types of breast cancer were enriched and separated by the DAVID database (**Table 1**). The results showed that there were some differences in the enrichment pathways of differential metabolic genes between the two types of breast cancer. We screened the first five pathways of the two types of breast cancer according to P value, in which purine metabolism, tyrosine metabolism, metabolism of xenobiotics by cytochrome P450, and drug metabolism were the main enrichment pathways of differential metabolic genes in HER2-positive breast cancer. The enrichment pathways of differential metabolic genes in triple-negative breast cancer mainly include purine metabolism, biosynthesis of antibiotics, pyrimidine metabolism, and metabolism of xenobiotics by cytochrome P450.

**Table 1:** KEGG pathway analysis about the differential metabolism-related genes in HER2-positive and triple-negative breast cancer.

	Terms	Count	PValue	FDR
HER2-positive	Metabolic pathways	199	3.99E-95	4.88E-92
	Purine metabolism	47	2.06E-26	2.52E-23
	Tyrosine metabolism	22	1.62E-21	1.98E-18
	Metabolism of xenobiotics by cytochrome P450	27	7.95E-19	9.70E-16
	Drug metabolism - cytochrome P450	25	1.63E-17	2.00E-14
Triple-negative	Metabolic pathways	230	9.34E-109	1.15E-105
	Purine metabolism	59	1.54E-35	1.89E-32
	Biosynthesis of antibiotics	50	2.64E-22	3.25E-19
	Pyrimidine metabolism	33	2.99E-19	3.68E-16
	Metabolism of xenobiotics by cytochrome P450	26	5.04E-16	6.88E-13

If there were more than 5 terms enriched in this category, top 5 terms were selected according to PValue. Count, the number of enriched genes in each term.

### Low Expression of Five Genes Is Associated With Increased Survival in Patients With HER2-Positive Breast Cancer

Changes in the expression of tumor-related metabolic genes affect disease progression, response to treatment, and functional consequences of patient survival. We analyzed the prognosis of abnormal expression of metabolism-related genes in HER2-positive breast cancer by univariate Cox method, and finally identified five genes significantly related to the survival of HER2-positive breast cancer patients, but it is worth noting that these five genes are not related to the survival of triple-negative breast cancer patients. These genes are *ATIC*, *HPRT1*, *ASNS*, *SULT1A2*, *HAL*. *ATIC*, *HAL*, *ASNS*, and *HPRT1* are enriched in purine metabolism and drug metabolism as well as other enzymatic pathways and *SULT1A2* is mainly enriched in the sulfur metabolism pathway. The following is a review of the relative expression of these genes in HER2-positive breast cancer and normal control tissues and their relationship with overall survival.

**Figure 2:**
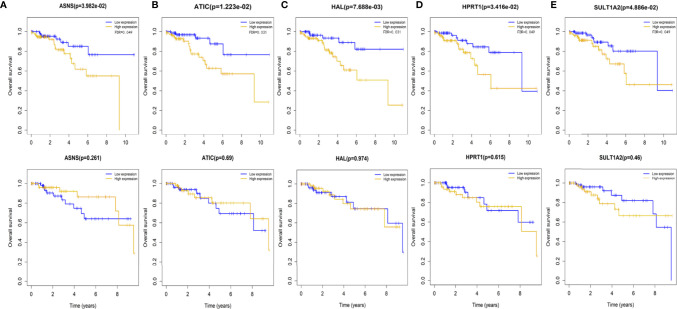
low expression of the five metabolism-related genes was associated with good survival in HER2-positive breast cancer patients, but not in triple-negative breast cancer patients. There were 156 positive cases of HER2-positive samples,114 cases of triple-negative samples. **(A)** ASNS **(B)** ATIC **(C)** HAL **(D)** HPRT1 **(E)** SULT1A2.

Compared with normal control tissues, the expression of *HPRT1*, *ASNS*, *ATIC*, *SULT1A2*, and HAL was significantly increased in HER2-positive breast cancer samples (**Table 2**). According to the median expression of these five genes, the overall survival time of patients with HER2-positive breast cancer was divided into two parts. We found that the low expression of the five genes was associated with good survival in HER2-positive breast cancer patients (**Figure 2**).

**Table 2:** Results of differential expression analysis of five metabolism-related genes.

	Gene	logFC	PValue	FDR
HER2-positive	ATIC	1.101524281	3.96E-29	6.31E-28
	HPRT1	1.124637282	5.95E-16	3.81E-15
	ASNS	1.047687465	2.03E-08	6.37E-08
	SULT1A2	1.177289372	0.000365503	0.000695067
	HAL	1.294131028	0.001153075	0.002031288
Triple-negative	HPRT1	1.678941692	4.73E-31	1.17E-29
	ASNS	1.915650172	1.81E-23	2.27E-22
	ATIC	0.931555679	3.64E-15	2.26E-14
	SULT1A2	-0.999901859	0.000228191	0.000446121
	HAL	-0.143030583	0.543223358	0.58463112

### Derivation of the HER2-Positive Breast Cancer Risk Scoring System

**Figure 3:**
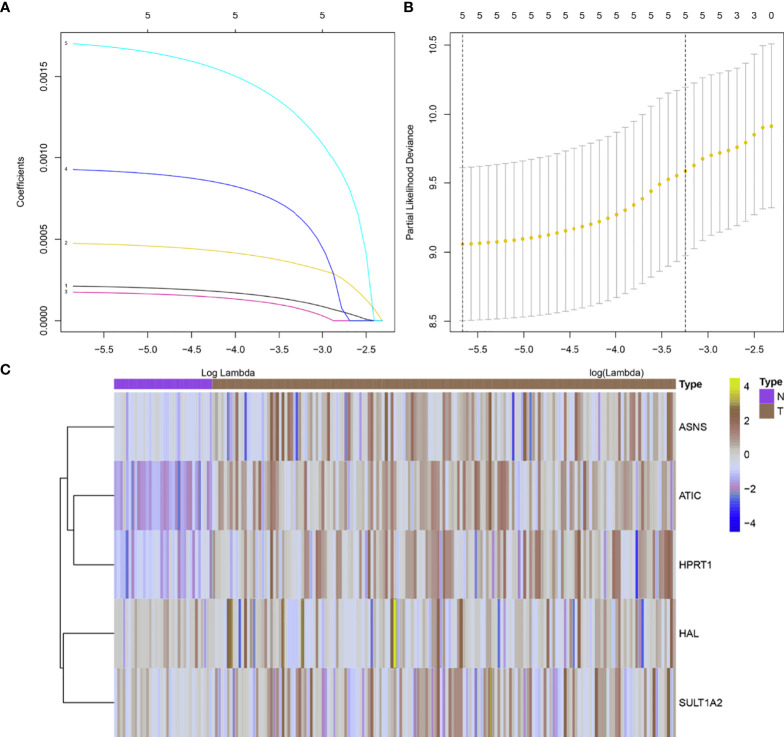
5 metabolism-related genes were identified for construction of the risk scoring system. **(A)** Validation was performed for tuning parameter selection through the LASSO regression model; **(B)** Elucidation for LASSO coefficient profiles of prognosis-related genes; **(C)** A heat map showing the expression of 5 metabolism-related genes. A total of 156 HER2-positive samples were analyzed here.

According to the results of the previous analysis, five abnormally expressed metabolic genes in survival-related HER2-positive breast cancer were identified for further construction of the risk scoring system. The lambda value with minimum error was determined by the cross-validation program to screen the genes that finally construct the risk scoring system. Through analysis, we found that the five abnormally expressed metabolic genes can be used as independent prognostic factors to construct the final metabolism-related risk scoring system for HER2-positive breast cancer (**Figure 3**). Based on the lasso Cox regression model, the risk score was determined for each sample based on the status of the 5 genes: Risk score = (0.000198599142304591 × *ATIC* expression) + (0.000454057389823286 × *HPRT1* expression) + (0.000161981657260441 × ASNS expression) + (0.000893755637962911 × *SULT1A2* expression) + (0.0016333682644294 × *HAL* expression).

**Figure 4:**
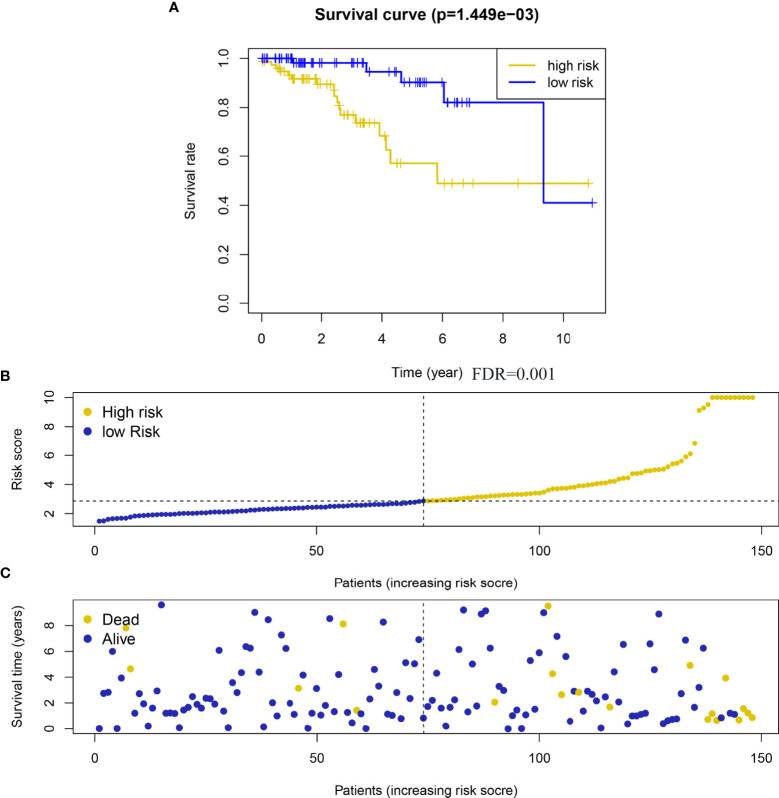
A risk scoring system was derived from the prognostic 5 metabolism-related genes. **(A)** Heatmap of the 5 metabolism-related genes expression in HER2-positive breast cancer. **(B)** The risk score distribution on the basis of the 5 metabolism-related genes; **(C)** The vital status of 156 patients with HER2-positive breast cancer in high‐ and low‐risk groups. A total of 156 HER2-positive samples were analyzed here.

Taking the median risk score as the critical value, 156 HER2-positive breast cancer samples with complete prognostic information were divided into high risk and low risk groups, and the OS of the high risk group was significantly shorter than that of the low risk group (**Figure 4**). In addition, the coefficients of the five genes defined by the lasso Cox regression model are all positive, indicating that these genes are closely related to the prognostic risk of HER2-positive breast cancer patients, and high expression corresponds to shorter OS.

### Comparative Analysis of Risk Scoring System and Clinical Factors

**Figure 5:**
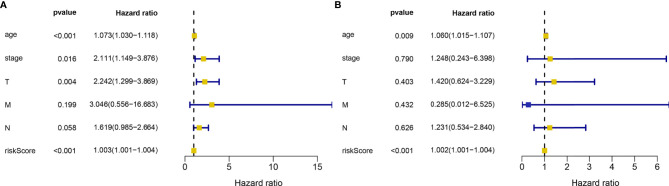
Univariate **(A)** and multivariate **(B)** analysis of risk score and clinical data related to HER2-positive breast cancer samples in TCGA database. The squares represent HR values, khaki means HR>1, blue means HR<1.The blue line represents the 95% confidence interval. A total of 133 HER2-positive samples were included in the analysis.

**Figure 6:**
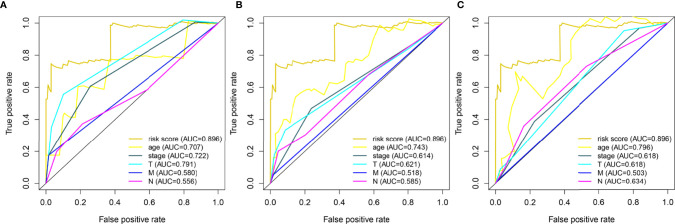
Time-dependent ROC curves for risk score and clinical data based on 5 metabolism-related genes. A total of 133 HER2-positive samples were included in the analysis. **(A)** 1 year-**(B)** 3 year-**(C)** 5year-.

Through the analysis using the lasso Cox regression model, it was found that the risk score system constructed by five metabolism-related genes could be used to predict the OS of HER2-positive patients. For further analysis, we included clinical factors in this study (**Table 3**). Univariate Cox analysis showed that risk score, age, stage, and T stage were closely related to OS in HER2-positive patients, which was statistically significant (**Figure 5A**). Multivariate Cox analysis showed that age and risk score could be used as independent prognostic factors to evaluate the survival rate of patients (**Figure 5B**). It can be seen that, whether using univariate analysis or multivariate analysis, our risk scoring system can effectively evaluate the prognosis, which further shows the evaluation value of the model. In order to further evaluate its predictive performance, we compared the predictive sensitivity of the risk scoring system with other clinical factors through the ROC curve and found that the predictive ability of the scoring system was higher than that of other clinical factors in predicting the 1-year, 3-year, or 5-year survival rate of patients (**Figure 6**).

**Table 3:** The specific baseline clinicopathological characteristics of 156 Her2-positive breast cancer samples.

	156 Her2-positive breast cancer samples
Age	
< =60 years	79
> 60 years	77
Stage	
I-II	110
III- IV	44
Unknown	2
Pathologic T stage	
T1	30
T2	103
T3-T4	23
Pathologic N stage	
N0-1	119
N2-3	35
Unknown	2
Pathologic M stage	
M0	132
M1	3
Unknown	21
Survival time	
< =3 years	99
> 3 years	57

## Discussion

In this study, we downloaded the RNA-SEQ data of HER2-positive breast cancer and triple-negative breast cancer from the TCGA database, then identified and compared the differential metabolic genes in HER2-positive, triple-negative, and normal adjacent control tissues and analyzed the pathway enrichment. Our results show that metabolic genes in HER2-positive breast cancer were significantly enriched in purine metabolism, metabolism of xenobiotics by cytochrome pathway, tyrosine metabolism and drug metabolism, indicating that HER2-positive breast cancer is significantly related to amino acid metabolic pathways and drug metabolism. while the metabolic genes in triple-negative breast cancer were significantly enriched in purine metabolism, metabolism of xenobiotics by cytochrome pathway. This may be related to the response of HER2-positive breast cancer on a variety of therapeutic modalities. In addition, we found that the low expression of five metabolic genes (*ATIC*, *HPRT1*, *ASNS*, *SULT1A2*, and *HAL*) was associated with significant improvement in the survival rate of HER2-positive patients. Based on results we propose that the products of these genes may be developed as therapeutic targets for the treatment of HER2-positive breast cancer. In addition, the prognostic risk prediction model constructed by these five genes could independently predict the prognosis of HER2-positive breast cancer patients, and could be used as a biomarker for patient prognosis.Therefore, on the basis of the current clinical prognosis data, the comprehensive use of the prognostic scoring system can help clinicians to better predict the prognosis of patients, so as to adopt more suitable intervention measures for patients, so as to implement individualized treatment for patients, better treat the patient’s condition.

*ATIC* catalyzes two steps of bifunctional enzymes in purine biosynthesis (aminoimidazole-4-carboxamide ribonucleotide converting enzyme/IMP cyclohydrolase) and participates in catalyzing the last two steps of purine biosynthesis from scratch (15). Uncontrolled proliferation is the hallmark of cancer that leads to an increased demand for *de novo* synthesis of purine and pyrimidine bases required for DNA and RNA biosynthesis. The purine biosynthetic pathway is often up-regulated in cancer (16). Our results suggest that limiting *ATIC* may provide a unique target for limiting purine biosynthesis in HER2-positive breast cancer. Antifolate Aminoimidazole-4-carboxamide Ribonucleotide Formyltransferase (AICARFT) is a folic acid-dependent catalytic site within the *ATIC* gene. LSN3213128 is a potent antifolate inhibitor of AICARFT. Treatment with this inhibitor in a mouse-based triple-negative breast cancer xenograft model showed inhibition of tumor growth (17). Considering that the experiments were performed using triple-negative breast cancer, but based on the correlations we observed between the expression of *ATIC* and the survival outcome of HER2-positive breast cancer, the targeted *ATIC* products may be more toxic to HER2-positive breast cancer.

*HPRT1* can transfer 5-phosphate ribosyl from 5-phosphate ribosyl pyrophosphate to purines, and plays an important role in purine nucleotide production through the purine rescue pathway. Our results show that low expression of *HPRT1* was associated with improved prognosis of HER2-positive breast cancer, which may be related to *HPRT1*’s involvement in the purine rescue pathway. Gedatolisib is a dual PI3K/mTOR inhibitor, and levels of mRNAs encoding HPRT1 (a key component of the purine rescue pathway) differ significantly between non-small cell lung cancer cells that are sensitive or resistant to gedatolisib. The resistance mechanism of PI3K pathway inhibitors is mediated by the activation of the purine rescue pathway, which provides a purine resource for tumor nucleotide biosynthesis (18). 8-azaguanine is an anti-metabolic drug activated by *HPRT1*. Increasing the gene dose of *HPRT1* in hyperploid breast cancer can control the sensitivity of this drug, which may be related to the function of *HPRT1* in purine biosynthesis (19). The relationship between *HPRT1* and drug treatment of HER2-positive breast cancer needs further exploration.

Asparagine synthetase (ASNS) is encoded by the asparagine synthetase (*ASNS*) gene and catalyzes the biosynthesis of L-asparagine (20). In our study, the low expression of *ASNS* in HER2-positive breast cancer is associated with patient survival, which may be due to ASNS-mediated asparagine synthesis in tumor cells. Asparagine synthetase is associated with apoptosis inhibition, protein biosynthesis, and *mTORC1* activation in non-small cell lung cancer, which inhibits ASNS expression, and, combined with the depletion of extracellular asparagine, can reduce the risk of non-small cell lung cancer growth (21). In cells with transient nuclear topoisomerase I downregulation, *ASNS* expression is reduced. In contrast, nuclear topology isomerase I complementary increased *ASNS*, suggesting that there is a causal relationship between nuclear topoisomerase I and *ASNS* expression, which needs to be further explored (22). Deprivation of endogenous *ASNS* expression can lead to inhibition of MDA-MB-231 cell proliferation, impaired colony formation, and impeded cell cycle progression. This may be because down-regulating *ASNS* expression can reduce the accumulation of pyrimidine bases in breast cancer cells, which is consistent with our findings that *ASNS* expression is elevated in triple-negative breast cancer (23). *ASNS* may be an attractive therapeutic target for HER2-positive breast cancer, and warrants further studies.

Sulfotransferase 1A2 (*SULT1A2*) mediates the metabolic activation of DNA-binding products by cancerous N-hydroxyarylamines. As a gene in the hormone metabolism and growth factor pathway, *SULT1A2* is related to breast density (24) and is involved in catechol estrogen metabolism (25). It is related to a variety of known risk factors for breast cancer carcinogenesis, and therefore may be a regulator of HER2-positive breast cancer risk. Patients with SULT1A2*2 and SULT1A2*3 alleles showed significantly higher plasma levels of 4-hydroxytamoxifen and endoxifen, and SULT1A2 appears to maintain optimal levels of 4-hydroxytamoxifen and endoxifen (26). Our results show that low expression of *SULT1A2* is associated with improved survival in HER2-positive breast cancer. The risk regulation of *SULT1A2* on HER2-positive breast cancer and its relevance to treatment need to be further explored.

Histidine ammonia lyase (*HAL*) is regulated by protein content and hormones (such as glucocorticoids and glucagon) in the diet (27). Histidine catabolism plays a key role in the formation and/or proliferation of certain stem cells, such as intestinal stem cells (28). Tumor cells are in a state of continuous division and proliferation, and amino acid metabolism is vigorous. The high expression level of *HAL* leads to a poor prognosis of estrogen receptor-positive breast cancer, which may be related to the involvement of *HAL* in amino acid metabolism and may be a potential therapeutic target (29). Our results show that low expression of *HAL* is associated with improved survival in HER2-positive breast cancer, suggesting that inhibition of tumor cell metabolism may be beneficial for tumor prognosis.

Based on the lasso Cox regression model, we constructed a risk scoring system and found that *ATIC*, *HPRT1*, *ASNS*, *SULT1A2*, and *HAL* were independently related to overall survival, indicating that collective changes in the expression of these five genes may be powerful predictors of clinical outcomes. Our results support this hypothesis, a total of 156 HER2-positive breast cancer samples with complete prognostic information were divided into high-risk and low-risk groups. The OS of the high-risk group was significantly shorter than that of the low-risk group. Therefore, the expression of these genes may have prognostic effects. Next, univariate and multivariate Cox analysis verified the predictive independence of the risk scoring system, and the predictive ability of the scoring system was higher than that of other clinical factors in predicting the 1-year, 3-year, or 5-year survival rate of patients. In addition, *ATIC*, *HPRT1*, *ASNS*, *SULT1A2*, and *HAL* may represent attractive therapeutic targets for HER2-positive breast cancer, and require further validation studies, especially considering that treatment with specific inhibitors may alter the expression of these metabolic genes.

Our study had a few limitations. Our breast cancer mRNA research data is derived from the TCGA database, but mRNA expression does not necessarily reflect protein expression levels (30). In addition, the RNA-seq data from the TCGA reflects the average mRNA expression in the entire tumor, but cannot recognize the difference in RNA expression among various tumor cells (that is, the expression differences between various tumor cells obtained from single-cell RNA sequencing platforms) (31). Finally, one must be aware that TCGA contains breast cancer data from a single, high-quality cohort, and it will be useful to verify these genes in other cohorts in the future. However, despite these limitations, the five metabolic genes identified in this study provide an interesting and reliable starting point and can provide new insights and directions for further study of disease mechanisms and treatment strategies at the metabolic level.

## Conclusion

Metabonomics can be used to identify novel prognostic markers or potential therapeutic targets. In the present study, a risk scoring system was constructed based on the expression data of metabolism-related genes in HER2-positive breast cancer. Patients were divided into different grades according to the expression of differential metabolic genes *ATIC*, *HPRT1*, *ASNS*, *SULT1A2*, and *HAL* in the system to evaluate the prognosis of patients. The products of these genes may be useful new therapeutic targets for HER2-positive breast cancer. Inhibition of the function of these metabolic genes can modulate the metabolism of tumors with low levels of metabolic gene expression, thus improving the survival rate of HER2-positive breast cancer patients.

## Data Availability Statement

The raw data supporting the conclusions of this manuscript will be made available by the authors, without undue reservation, to any qualified researcher.

## Ethics Statement

Ethical review and approval was not required for the study on human participants in accordance with the local legislation and institutional requirements. Written informed consent for participation was not required for this study in accordance with the national legislation and the institutional requirements.

## Author Contributions

CG and CS conceived and designed the study; JZ and CL performed data analysis; CZ and LL contributed analysis tools; CG and HL wrote the paper. All authors contributed to the article and approved the submitted version.

## Funding

This work is supported by the grants from National Natural Science Foundation of China (81973677) and National Natural Science Foundation of China (82174222).

## Conflict of Interest

The authors declare that the research was conducted in the absence of any commercial or financial relationships that could be construed as a potential conflict of interest.

## Publisher’s Note

All claims expressed in this article are solely those of the authors and do not necessarily represent those of their affiliated organizations, or those of the publisher, the editors and the reviewers. Any product that may be evaluated in this article, or claim that may be made by its manufacturer, is not guaranteed or endorsed by the publisher.
